# Tedizolid Activity Against Clinical *Mycobacterium abscessus* Complex Isolates—An *in vitro* Characterization Study

**DOI:** 10.3389/fmicb.2018.02095

**Published:** 2018-09-07

**Authors:** Ying Wei Tang, Bernadette Cheng, Siang Fei Yeoh, Raymond T. P. Lin, Jeanette W. P. Teo

**Affiliations:** ^1^Department of Biological Sciences, National University Singapore, Singapore, Singapore; ^2^Department of Laboratory Medicine, National University Hospital, Singapore, Singapore; ^3^Pharmacy, National University Hospital, Singapore, Singapore; ^4^National Public Health Laboratory, Ministry of Health, Singapore, Singapore

**Keywords:** multidrug resistant (MDR), inducible resistance, oxazolidinone, time-kill, repurposable drugs

## Abstract

*Mycobacterium abscessus* complex consist of three rapidly growing subspecies: *M. abscessus, M. massiliense*, and *M. bolletii*. They are clinically important human pathogens responsible for opportunistic pulmonary and skin and soft tissue infections. Treatment of *M. abscessus* infections is difficult due to *in vitro* resistance to most antimicrobial agents. Tedizolid (TZD) is a next-generation oxazolidinone antimicrobial with a wide spectrum of activity even against multidrug resistant Gram-positive bacteria. In this study, the *in vitro* activity of TZD against the *M. abscessus* complex (*n* = 130) was investigated. Susceptibility testing by broth microdilution showed lower TZD minimum inhibitory concentrations (MICs) when compared to linezolid. The MIC_50_ and MIC_90_ was 1 mg/L and 4 mg/L, respectively across all *M. abscessus* complex members, reflecting no difference in subspecies response to TZD. Pre-exposure of *M. abscessus* complex to subinhibitory concentrations of TZD did not trigger any inducible drug resistance. Single-drug time kill assays and bactericidal activity assays demonstrated bacteriostatic activity of TZD in all three *M. abscessus* subspecies, even at high drug concentrations of 4 to 8x MIC. Combination testing of TZD with clarithromycin, doxycycline and amikacin using the checkerboard approach showed no antagonistic interactions. TZD may be an effective therapeutic antimicrobial agent for the treatment of *M. abscessus* infections.

## Introduction

*Mycobacterium abscessus* complex consists of three rapidly-growing mycobacteria (RGM) subspecies: *M. abscessus* subspecies *abscessus, M. abscessus* subspecies *massiliense* and *M. abscessus* subspecies *bolletii* (Lee et al., 2015). They have emerged as clinically important multi-drug resistant (MDR) human pathogens responsible for a wide spectrum of skin and soft tissue infections (SSTIs), opportunistic infections in immunocompromised patients and pulmonary infections in patients with chronic pulmonary disease or cystic fibrosis (Nessar et al., 2012). Nosocomial outbreaks of *M. abscessus* have been reported worldwide, highlighting its clinical significance (Nessar et al., 2012). *M. abscessus* complex accounts for approximately 65–80% of pulmonary infections caused by RGM (Koh et al., 2011). In Singapore, *M. abscessus* complex is the most prevalent RGM isolated in hospitals and accounts for approximately 35% of all non-tuberculous mycobacteria (NTM) infections (Tang et al., 2015).

*M. abscessus* pulmonary infections are infamously difficult to treat, with low cure rates ranging from 30 to 50%. This is attributed to natural resistance to most antimicrobial agents (Van Ingen et al., 2012). Existing treatment regimens are combination-based therapies usually consisting of a macrolide antibiotic such as clarithromycin (CLR), amikacin (AMK) and either cefoxitin (FOX), imipenem (IPM), or tigecycline (TGC) (Van Ingen et al., 2012). The administration of combination therapy (usually CLR and AMK) is lengthy, lasting for periods of between 2 and 4 months before clinical and microbiological improvements are noticeable (Huang et al., 2010). And the lack of alternative antimicrobial options further complicates the treatment of NTM infections (Benwill and Wallace, 2014).

Tedizolid (TZD) is a next-generation oxazolidinone antibiotic approved by the Food and Drug Administration (FDA) in 2014 for the treatment of acute bacterial skin and skin structure infections (ABSSSI) caused by certain *Streptococcus* spp. and methicillin-resistant *Staphylococcus aureus* (MRSA). Phase three clinical trials demonstrated non-inferiority of TZD to the first-in-class oxazolidinone LZD for the treatment of ABSSI, with improved clinical efficacy against MRSA and slightly improved safety profile (Moran et al., 2014). Oxazolidinones are protein synthesis inhibitors (Rybak et al., 2014) whose action is primarily bacteriostatic (Rybak et al., 2014). *In vitro*, TZD has demonstrated activity against acid-fast bacilli such as slow-growing *Mycobacterium tuberculosis* and the rapidly-growing *Mycobacterium fortuitum* (Kisgen et al., 2014). TZD MIC values against NTM were equivalent or 1- to 8-fold lower than those of LZD, indicating improved *in vitro* potency (Brown-Elliott and Wallace, 2017). Another study showed that TZD exhibited good bacteriostatic activity against *M. abscessus*, with MICs two- to 16-fold lower as compared to LZD (Compain et al., 2018). The combination of *in vitro* activity against MDR Gram-positive bacteria, an oral dosage formulation and once-daily dosing makes TZD a promising investigational antimicrobial therapeutic agent (Kisgen et al., 2014).

In this study, we explored the potential use of TZD for anti-mycobacterial therapy by characterizing the *in vitro* activity of TZD against 130 clinical isolates of *M. abscessus* complex members.

## Materials and methods

### Mycobacterial isolates and genetic characterization

A total of 130 retrospective non-duplicate clinical *M. abscessus* complex isolates were evaluated. This collection consisted of 43 *M. abscessus* isolates, 82 *M. massiliense* isolates and five *M. bolletii* isolates. The subspecies of the *M. abscessus* complex isolates was determined by multi-locus sequencing employing the *rpoB* and *hsp65* genes (Macheras et al., 2011). CLR resistance was analyzed by full-length sequencing of the *erm*(41) and *rrl* genes (Aziz et al., 2017). For *erm*(41), the full-length 673 bp gene sequence was examined for T/C polymorphism at the 28th nucleotide position as well as for gene deletions. *erm*(41) T28 sequevars have wild-type inducible CLR resistance whilst C28 sequevars are phenotypically CLR susceptible (Choi et al., 2012). For the *rrl* gene, the nucleotides 2058–2059, associated with CLR resistance were examined.

### MIC determination

Antibiotic powders of TZD, CLR, and LZD were purchased from MedChem Express (NJ, USA). Antimicrobial susceptibility testing of TZD, CLR and LZD were performed using the microdilution method according to the Clinical & Laboratory Standards Institute (CLSI) guidelines (CLSI, 2015). The working range for all tested antimicrobials was 0.125–64 mg/L. For TZD and LZD, the inoculated microdilution plates were incubated at 30°C for 3–5 days before growth was assessed by visual inspection. The MIC was determined as the concentration of antibiotic at which there was no visible growth. *Staphylococcus aureus* ATCC (American type culture collection) 6538 and *Enterococcus faecalis* ATCC 29212 were used as susceptibility testing quality control strains. The MIC for the control strains fell within the acceptable MIC range of 0.25–1 mg/L for both TZD and LZD (Woods et al., 2011; Brown-Elliott and Wallace, 2017). For TZD, there are currently no interpretative criteria for RGM. For LZD, RGM with MICs of ≤8 were classified as sensitive and ≥32 as resistant (Woods et al., 2011).

### Bactericidal/static activity determination

For the bactericidal/static activity determination assay, *M. abscessus* isolates (*n* = 7), *M. massiliense* isolates (*n* = 15) and *M. bolletii* (*n* = 5) were tested. After three days of TZD incubation at 30°C, the entire 96-well microtiter plate well contents corresponding to the two-fold diluted TZD concentrations (64–0.0625 mg/L) were plated and the CFU determined. The Minimum Bactericidal Concentration (MBC) of TZD against the tested isolates was defined as the lowest drug concentration required to induce ≥99.9% cell death as compared to the untreated control at the 0 h time point. For bactericidal antibiotics, the MBC is classified as ≤ 4 times the MIC while the MBC is usually >4 times the MIC for bacteriostatic antibiotics.

### TZD time kill assay for the *M. abscessus* complex

Time-kill assays were performed according to CLSI guidelines (CLSI, 1999) and were setup for a single isolate each of *M. abscessus, M. massiliense* and *M. bolletii* using a 10^6^ CFU/mL inoculum exponential growth phase bacterial suspension. Two-fold increasing concentrations of TZD (from 0.25 to 8x MIC) and a drug-free growth control was used. At time intervals of 0, 4, 8, 12, 24, 36, 48, 72, 96, and 120 h CFU enumerations were made. Bactericidal activity was defined as a ≥3-log_10_ decrease in CFU/mL at 120 h when compared to the 0 h time point. All time-kill experiments were performed in duplicate and the mean CFU counts plotted.

### TZD pre-exposure assay

*erm*(41) confers inducible macrolide resistance in the *M*. *abscessus* complex, observable phenotypically at day 14 of incubation (Rubio et al., 2015). To examine if a similar inducible phenomenon existed for TZD, *M. abscessus* complex isolates were pre-exposed to sub-inhibitory concentrations of TZD prior to MIC determination as previously described (Aziz et al., 2017). TZD pre-exposure assays were performed for three isolates each of *M. abscessus, M. massiliense* and *M. bolletii*. Briefly, 10^6^ CFU/mL bacterial suspension was treated with TZD at a sub-inhibitory concentration of 0.25 mg/L for *M. abscessus* and *M. massiliense* isolates, and at 1 mg/L for *M. bolletii* isolates, four-fold lower than their MIC_50_ values. An untreated, drug-free culture was setup as a growth control. The MICs were determined at day 3 and at day 14.

### Synergy studies using checkerboard titration assay

The *in vitro* interactions of TZD and CLR, TZD and DOX, as well as TZD and AMK were investigated by the checkerboard approach using the broth microdilution method as previously described (Kaushik et al., 2015). Five isolates of *M. abscessus*, four *M. massiliense* and five *M. bolletii* isolates were used for evaluation. The fractional inhibitory concentration index (∑FIC) for each isolate was calculated as follows: $\sum \text{FIC}=\;\frac{MIC\;of\;antibiotic\;1\;in\;combination}{MIC\;of\;antibiotic\;1\;only}+\;\frac{MIC\;of\;antibiotic\;2\;in\;combination}{MIC\;of\;antibiotic\;2\;only}$. Synergy was defined as a FIC index of ≤0.5, indifference by a FIC index of >0.5 to ≤4 and antagonism when the FIC index was >4.

## Results

### Susceptibility of *M. abscessus* complex isolates to tedizolid, clarithromycin, and linezolid

For TZD, the MIC range was 0.0625–8 mg/L, compared to 0.0625–>32 mg/L for LZD. The MIC_50_ and MIC_90_ for TZD was 1 and 4 mg/L, consistent across all three subspecies suggesting that the 3 subspecies were similarly responsive to TZD. In general, the TZD MICs were 2- to 16-fold lower than those of LZD. Due to the lack of interpretive criteria for TZD for RGM, susceptibility rates were not assigned (Table 1). For LZD, 52.3% of all isolates were susceptible (MIC ≤ 8 mg/L); 53.5% of *M. abscessus*, 20% of *M. bolletii* and 53.7% of *M. massiliense* (Table 1).

**Table 1:** MICs of tedizolid, clarithromycin and linezolid for 130 clinical isolates of *Mycobacterium abscessus* complex.

**Antimicrobial agent**	***M. abscessus* complex (*n* = 130)**	**MIC_50_**	**MIC_90_**	**MIC range (mg/L)**	Susceptibility (%)^*^
Tedizolid	*M. abscessus* (43)	1	4	0.0625–8	N/A
	*M. bolletii* (5)	4	4	1–8	N/A
	*M. massiliense* (82)	1	4	0.0625–8	N/A
	Total (130)	1	4	0.0625–8	N/A
Linezolid	*M. abscessus* (43)	8	>32	0.0625–>32	53.5
	*M. bolletii* (5)	32	>32	8– >32	20
	*M. massiliense* (82)	8	>32	0.5–>32	53.7
	Total (130)	8	>32	0.0625–>32	52.3
Clarithromycin^*^	*M. abscessus* (43)	>16	>16	0.0625–>16	20.9
	*M. bolletii* (5)	>16	>16	1–>16	40
	*M. massiliense* (82)	0.5	12	0.0625–>16	76.8
	Total (130)	1	>16	0.0625–>16	55.4

* *For LZD, isolates with MICs of ≤8 were classified as sensitive and ≥32 were resistant (Woods et al., 2011)*.

*For CLR, isolates with MICs of ≤2 were classified as sensitive and ≥8 were resistant*.

For CLR, the MIC_50_ and MIC_90_ were both >16 mg/L for *M. abscessus* and *M. bolletii*, as compared to 1 and 12 mg/L for *M. massiliense*. According to CLSI interpretive criteria for susceptibility (CLSI, 2017), 55.4% (72/130) of all isolates were susceptible to CLR (MIC < 2 mg/L). *M. massiliense* isolates showed susceptibility rates of 76.8%. This is consistent with the observation that *M. massiliense* usually possesses a truncated non-functional *erm*(41) (Chew et al., 2017). In contrast, *M. abscessus* (20.9% susceptible) and *M. bolletii* isolates (0% susceptible) showed higher rates of resistance to CLR. The susceptible *M. abscessus* isolates were *erm*(41) C28 sequevar.

### TZD does not exhibit bactericidal activity against the *M. abscessus* complex

**Figure 1:**
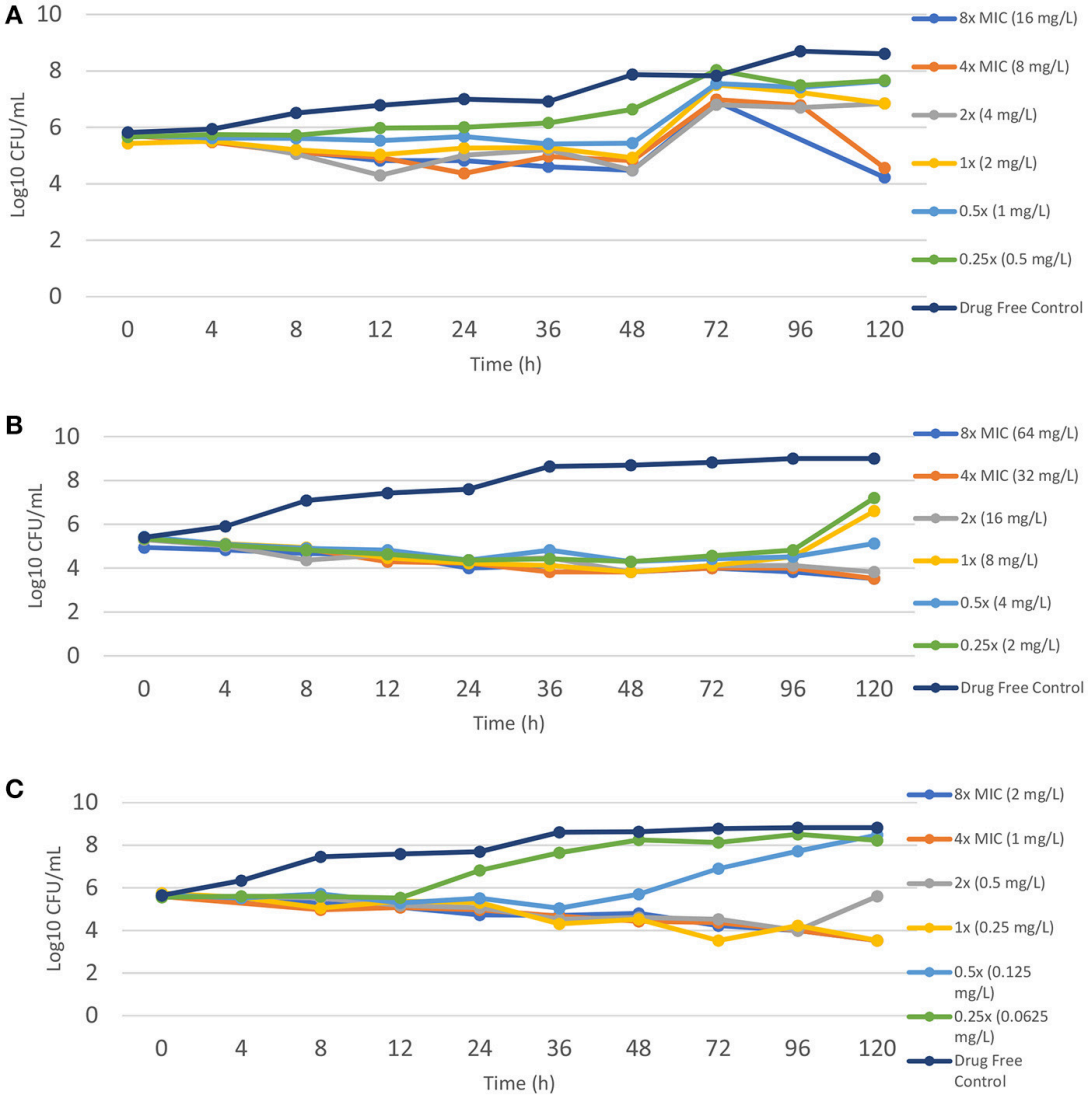
Time kill kinetics of tedizolid against different *M. abscessus* subspecies **(A)** *M. abscessus* (TZD MIC = 2 mg/L); **(B)** *M. bolletii* (TZD MIC = 8 mg/L); *M. massiliense* (TZD MIC = 0.5 mg/L). Each point represents the mean of duplicate determinations.

Time kill assays were performed using TZD for one isolate each of *M. abscessus* (MIC = 2 mg/L), *M. bolletii* (MIC = 8 mg/L) and *M. massiliense* (MIC = 0.25 mg/L). TZD did not exhibit bactericidal activity in all three subspecies, even at concentrations of 4- and 8-fold higher than the MIC determined by the microdilution method (Figure 1). There was a general decline in CFU count over time for *M. bolletii* and *M. massiliense*. Bacterial regrowth (0.2 log_10_CFU/ml greater than the starting inoculum) was observed at time point 72 h for *M. abscessus* for all drug concentrations (0.5–8x MIC), following which a reduction in CFU was only observed at concentrations of 4x and 8x MIC. For *M. bolletii*, regrowth was noted at time point 120 h for concentrations of 0.25x and 1x MIC. Regrowth was observed at time point 12 h for 0.25x MIC and 72 h for 0.5x MIC for *M. massiliense* (Figure 1).

### TZD exhibits bacteriostatic activity against *M. abscessus* complex

TZD exhibited bacteriostatic activity against all tested isolates of the *M*. *abscessus* complex (Table 2). The MBC of all three subspecies was greater than four times of MIC, which is characteristic of a bacteriostatic antimicrobial agent.

**Table 2:** Determination of the MBC and antibacterial mode of tedizolid against the *Mycobacterium abscessus* complex.

***M. abscessus* complex**	**MBC range (mg/L)**	**Median MBC (mg/L)**	**MIC range (mg/L)**	**Median MIC (mg/L)**	**Mode of action**
*M. abscessus* (*n* = 7)	>64	>64	0.125–8	4	Bacteriostatic
*M. bolletii* (*n* = 5)	8–>64	32	1–8	4	Bacteriostatic
*M. massiliense* (*n* = 15)	16–>64	>64	0.125–8	0.5	Bacteriostatic

### TZD pre-exposure does not induce resistance

Pre-treatment of *three M. abscessus, M. bolletii* and *M. massiliense* isolates to sub-inhibitory concentrations of TZD did not affect MICs (Table 3). MICs after pre-exposure to TZD were similar to those without pre-exposure, suggesting that *M. abscessus* did not harbor inducible TZD resistance mechanisms.

**Table 3:** MICs of *Mycobacterium abscessus* complex after exposure to sub-inhibitory concentrations of tedizolid.

*M. abscessus* complex^*^	**TZD MIC (mg/L)**	
	**No pre-exposure**	**After pre-exposure**
*M. abscessus* #1	4	2
*M. abscessus* #2	4	2
*M. abscessus* #3	8	4
*M. bolletii* #1	0.5	1
*M. bolletii* #2	0.5	0.5
*M. bolletii* #3	4	2
*M. massiliense* #1	4	2
*M. massiliense* #2	4	4
*M. massiliense* #3	4	4

* *Three unique isolates of each subspecies were used for testing*.

### Checkerboard testing of TZD in combination with clarithromycin, doxycycline and amikacin suggests interactions that are largely indifferent

Amikacin (AMK) and clarithromycin (CLR) are currently the only two antimicrobial agents with reliable *in vitro* activity against *M. abscessus* (Tang et al., 2015). TZD in combination with CLR, DOX and AMK were evaluated for antimicrobial activity against five isolates of *M. abscessus*, four isolates of *M. bolletii* and five isolates of *M. massiliense* by checkerboard synergy approach (Table 4). No instances of antagonism were observed in any antimicrobial combination tested. Indifference was the primary interaction accounting for 90.5% of all interactions. For the combination of TZD and CLR, all interactions were indifferent. One instance of synergistic interaction was observed in the *erm*(41) C28 sequevar *M. bolletii* isolate for the combination of TZD and DOX. In two *erm*(41) T28 sequevar *M. abscessus* isolates and one *M. massiliense* isolate, synergistic interactions were observed for the combination of TZD and AMK (Table 4). Overall, the findings suggest that TZD has no interaction when used in combination with CLR, DOX and AMK against *M. abscessus*.

**Table 4:** FIC index for tedizolid tested in combination with clarithromycin, doxycycline and amikacin against the *Mycobacterium abscessus* complex.

			**MIC (mg/L)**				**FIC index^#^**		**FIC index^#^**		**FIC index^#^**	
***M. abscessus* complex**	**Susceptibility to CLR**	***erm*(41)^$^**	**TZD**	**CLR**	**DOX**	**AMK**	**TZD + CLR**	**Interaction**	**TZD + DOX**	**Interaction**	**TZD + AMK**	**Interaction**
*M. abscessus* (*n* = 1)	S	C28 sequevar	1	2	1	1	1.000	Indifference	1.000	Indifference	0.625	Indifference
*M. abscessus* (*n* = 4)	R	T28 sequevar	3	>16	1	1	1.000	Indifference	1.000	Indifference	0.625	Indifference
*M. abscessus*	R	T28 sequevar	4	>16	1	16	1.016	Indifference	0.625	Indifference	0.375	Synergistic
*M. abscessus*	R	T28 sequevar	1	>16	1	2	1.016	Indifference	1.000	Indifference	1.063	Indifference
*M. abscessus*	R	T28 sequevar	8	>16	1	2	1.016	Indifference	1.125	Indifference	0.750	Indifference
*M. bolletii* (*n* = 1)	S	C28 sequevar	1	1	8	16	1.016	Indifference	0.313	Synergistic	0.53	Indifference
*M. bolletii* (*n* = 3)	R	T28 sequevar	8	>16	2	16	1.016	Indifference	0.563	Indifference	1.008	Indifference
*M. bolletii*	R	T28 sequevar	4	>16	1	8	1.500	Indifference	0.625	Indifference	0.563	Indifference
*M. bolletii*	R	T28 sequevar	4	>16	4	0.5	1.016	Indifference	0.625	Indifference	0.750	Indifference
*M. massiliense* (*n* = 5)	S	Deleted	0.25	0.25	2	2	1.016	Indifference	0.563	Indifference	0.625	Indifference
*M. massiliense*	S	Deleted	2	2	1	1	2.016	Indifference	1.000	Indifference	0.750	Indifference
*M. massiliense*	S	Deleted	1	1	2	2	0.516	Indifference	0.625	Indifference	0.375	Synergistic
*M. massiliense*	S	Deleted	0.125	0.125	1	2	1.016	Indifference	0.750	Indifference	0.750	Indifference
*M. massiliense*	S	Deleted	1	1	1	1	1.016	Indifference	0.750	Indifference	1.000	Indifference

$ *T28 sequevar are CLR resistant, C28 sequevars are CLR susceptible. Deleted, refers to 274 bp erm(41) gene deletion characteristic in the M. massiliense subspecies*.

# *FIC index was calculated as [(MIC of tedizolid in combination/MIC of tedizolid alone) + (MIC of second antibiotic in combination/MIC of second antibiotic alone)]*.

*Only FIC index <0.5 was considered as a synergistic interaction*.

## Discussion

*M. abscessus* pulmonary infections are notoriously difficult to treat with low cure rates of 30–50% (5). This has spurred drug repurposing, defined as the “off-label” usage of existing antimicrobials (Palomino and Martin, 2014). LZD was initially developed for the treatment of infections caused by β-lactam-resistant Gram-positive bacteria, but it is now a recommended second-line drug for the treatment of MDR and extensively drug-resistant tuberculosis (Dheda et al., 2017). Furthermore, TZD has demonstrated *in vitro* activity against mycobacterial pathogens such as *Mycobacterium tuberculosis* and *Mycobacterium fortuitum* (Kisgen et al., 2014).

In our study, the potential of TZD for the treatment of *M. abscessus* complex infections was investigated *in vitro*. In comparison to two recent studies, our TZD MIC range of 0.0625–8 mg/L (MIC_50_ = 1 mg/L, MIC_90_ = 4 mg/L) for 43 *M. abscessus* isolates was lower than the MIC range of 0.12–>32 μg/mL (MIC_50_ = 4 μg/mL, MIC_90_ = 8 μg/mL) reported by Brown-Elliott and Wallace (2017) and the MIC range of 1–16 μg/mL (MIC_50_ = 2 μg/mL, MIC_90_ = 8 μg/mL) reported by Compain et al. (2018). For the 82 *M. massiliense* isolates a TZD MIC range of 0.0625–8 mg/L (MIC_50_ = 1 mg/mL, MIC_90_ = 4 mg/mL) was obtained. Brown-Elliott & Wallace Jr. reported a TZD MIC range of 0.12–>32 μg/mL (MIC_50_ = 2 μg/mL, MIC_90_ = 4 μg/mL) for a smaller set of 12 isolates whilst Compain et al. reported a MIC range of 1–8 μg/mL (MIC_50_ = 4 μg/mL, MIC_90_ = 8 μg/mL) for 14 *M. massiliense* isolates (n = 14). The TZD MIC range of 1–8 mg/L (MIC_50_ and MIC_90_ = 4 mg/L) for 5 *M. bolletii* isolates were comparable to the MIC range of 1–4 mg/L (MIC_50_ = 2 μg/mL, MIC_90_ = 4 μg/mL) as determined by Compain et al. (2018).

There are currently no CLSI recommended TZD breakpoints for Mycobacteria but LZD is considered a reliable surrogate antimicrobial agent for TZD susceptibility, with the European Committee on Antimicrobial Susceptibility Testing (EUCAST) recommending the reporting of isolates susceptible to LZD as also susceptible to TZD (EUCAST, 2016). LZD susceptibility was found to be highly predictive of TZD susceptibility, with high categorical agreement between MIC values of LZD and TZD, and low rates of very major errors for Gram-positive bacteria (e.g., *Staphylococcus* spp. and *Enterococcus* spp.) (Zurenko et al., 2014). All 130 *M. abscessus* isolates were susceptible to TZD when a breakpoint ≤ 8 mg/L was applied. Although the suitability of LZD as a surrogate for TZD susceptibility has only been recommended for Gram-positive bacteria, these findings suggest that *M. abscessus* may be more susceptible to TZD than LZD.

In *M. tuberculosis* oxazolidinone resistance is associated with point mutations in the 23S rRNA gene (*rrl*) and in the 50S ribosomal protein L3 (Klitgaard et al., 2015; McNeil et al., 2017). *rrl* and L3 mutant strains were resistant to LZD and cross-resistant to sutezolid, a next-generation oxazolidinone currently in clinical development with improved potency against *M. tuberculosis* (McNeil et al., 2017). Furthermore, Gram-positive bacteria with 23S rRNA gene mutations were found to have high LZD (16 mg/L) and TZD MICs (>1 mg/L) as reported in a 2011–2012 surveillance report of TZD activity (Bensaci and Sahm, 2017). In our study, 5 isolates with high TZD MIC (8 mg/L) had their full–length *rrl* gene sequenced. All the sequenced isolates possessed a wild type *rrl* gene. This suggests alternative resistance mechanisms, such as efflux pumps (Gupta et al., 2006). We acknowledge our study limitation where mutations in bacterial 50S ribosomal protein L3, which are associated with oxazolidinone resistance, were not investigated.

This is currently the first study (to the best of our knowledge) to perform a time-kill assay for all three subspecies of the *M. abscessus* complex. TZD exhibits little concentration-dependent killing and no significant bactericidal activity against the three subspecies at all tested drug concentrations (0.5x−8x MIC). Compain et al. reported similar time-kill kinetics for *M. abscessus* ATCC 19977/CIP 104536, with no bactericidal activity at TZD concentrations of 4 and 8 mg/L. Bacterial regrowth was observed in *M. abscessus* in the logarithmic phase of growth for TZD concentrations tested. In comparison, regrowth was only observed at lower TZD concentrations of 0.25x and 1x MIC for *M. bolletii* and 0.25x and 0.5x MIC for *M. massiliense*. These findings were consistent with the findings by Ferro et al. (2015) where regrowth was also observed for AMK and CLR after 72 hours even at concentrations of 2x to 8x MIC. The findings suggest that a TZD concentration of ≥4x MIC may be required to induce significant killing activity against *M. abscessus*, whereas a TZD concentration of 1x/2x MIC sufficiently reduces bacterial counts in *M. bolletii* and *M. massiliens*e over time. Similar to other active antimicrobials against *M. abscessus* complex, TZD exhibits a bacteriostatic effect that is more pronounced in *M. bolletii* and *M. massiliense* than *M. abscessus*.

Synergy studies of TZD with CLR, DOX, AMK demonstrated that all combinations primarily showed indifferent interactions with no instances of antagonism. A similar study performed by Compain et al. reported indifferent interactions of TZD with TGC, AMK and ciprofloxacin, with the combination of CLR and TZD showing one synergistic interaction out of 6 tested isolates. The findings suggest that TZD could be used in the existing combination regime of CLR and AMK with no antagnostic interactions.

## Author contributions

YT drafted the manuscript and performed the experiments. BC performed the experiments. SY provided clinical feedback and scientific review. RL provided clinical feedback and scientific review. JT drafted the manuscript and oversaw the project execution.

### Conflict of interest statement

The authors declare that the research was conducted in the absence of any commercial or financial relationships that could be construed as a potential conflict of interest. The handling editor declared a past co-authorship with one of the authors JT.
